# A Unique Mono- and Diacylglycerol Lipase from *Penicillium cyclopium*: Heterologous Expression, Biochemical Characterization and Molecular Basis for Its Substrate Selectivity

**DOI:** 10.1371/journal.pone.0102040

**Published:** 2014-07-22

**Authors:** Zhong-Biao Tan, Jian-Fang Li, Xue-Ting Li, Ying Gu, Min-Chen Wu, Jing Wu, Jun-Qing Wang

**Affiliations:** 1 Key Laboratory of Carbohydrate Chemistry and Biotechnology, Ministry of Education, School of Biotechnology, Jiangnan University, Wuxi, China; 2 School of Food Science and Technology, Jiangnan University, Wuxi, China; 3 Wuxi Medical School, Jiangnan University, Wuxi, China; Concordia University Wisconsin, United States of America

## Abstract

A cDNA gene encoding a mature peptide of the mono- and diacylglycerol lipase (abbreviated to PcMdl) from *Penicillium cyclopium* PG37 was cloned and expressed in *Pichia pastoris* GS115. The recombinant PcMdl (rePcMdl) with an apparent molecular weight of 39 kDa showed the highest activity (40.5 U/mL of culture supernatant) on 1,2-dibutyrin substrate at temperature 35°C and pH 7.5. The rePcMdl was stable at a pH range of 6.5–9.5 and temperatures below 35°C. The activity of rePcMdl was inhibited by Hg^2+^ and Fe^3+^, but not significantly affected by EDTA or the other metal ions such as Na^+^, K^+^, Li^+^, Mg^2+^, Zn^2+^, Ca^2+^, Mn^2+^, Cu^2+^, and Fe^2+^. PcMdl was identified to be strictly specific to mono- and diacylglycerol, but not triacylglycerol. Stereographic view of PcMdl docked with substrate (tri- or diacylglycerol) analogue indicated that the residue Phe^256^ plays an important role in conferring the substrate selectivity. Phe^256^ projects its side chain towards the substrate binding groove and makes the *sn*-1 moiety difficult to insert in. Furthermore, *sn*-1 moiety prevents the phosphorus atom (substitution of carboxyl carbon) from getting to the O_γ_ of Ser^145^, which results in the failure of triacylglycerol hydrolysis. These results should provide a basis for molecular engineering of PcMdl and expand its applications in industries.

## Introduction

Lipases (triacylglycerol hydrolases, EC 3.1.1.3), one of serine hydrolases, catalyze the hydrolysis of triacylglycerols into diacylglycerols, monoacylglycerols, glycerols and free fatty acids [1]. Mono- and diacylglycerol lipases (Mdls) are defined as the enzymes capable of hydrolyzing mono- and diacylglycerols but not triacylglycerols [2]. The Mdl from *Penicillium camembertii* can hydrolyze 1-rac-monoolein, 2-monoolein, 1,3-diolein, and 1,2-rac-diolein but not triolein [3]. According to the results of searches in GenBank (http://www.ncbi.nlm.nih.gov/), microbes which can produce Mdl are only limited in a few genuses such as *Aspergillus*, *Penicillium*, *Malassezia*, and so on.

The correlation between structure and function of Mdl has been studied widely. By site-directed mutagenesis, it was confirmed that the Mdl from *P. camembertii* has a similar catalytic center, Ser-Asp-His, to that of a triacylglycerol lipase family [3]. Analysis of the binding of diacylglycerol analogue to the Mdl from *Malassezia globosa* suggested that the substrate specificity of the enzyme mainly resulted from the shape and size of a narrow tunnel, in which there was no space for the settlement of the third chain of triacylglycerol [4], [5]. The *mdl* from *P. camembertii* U-150 was heterologously expressed in *Saccharomyces cerevisiae* and *Aspergillus oryzae* [3], [6]. However, there are few reports concerning the heterologous expression of *mdl* in *Pichia pastoris* and the molecular basis for substrate selectivity of Mdl from genus *Penicillium*.

In our previous studies, the genomic DNA encoding a mono- and diacylglycerol lipase (PcMdl) was cloned (GenBank: AF288219) from *Penicillium cyclopium* PG37, which can produce PcMdl together with an alkaline lipase (Lip I). The *lip* I gene was efficiently expressed in *P. pastoris* GS115 by induction under optimized conditions [7]. In this work, the cDNA encoding PcMdl was cloned and functionally expressed in *P. pastoris*. The character of the recombinant PcMdl (rePcMdl), as well as the molecular basis for substrate selectivity of PcMdl was investigated.

## Materials and Methods

### Ethics Statement

No specific permissions were required for these locations/activities. The field studies did not involve endangered or protected species and the specific location of this study is longitude: 120.27E, latitude: 31.58N.

### Strains, Vectors, and Culture Media

*P. cyclopium* PG37, isolated from the soil in China and deposited in the Lab of Biochemistry and Molecular Biology, Wuxi Medical School, Jiangnan University, China, was used as the donor of a gene *Pcmdl*. The strain was cultured in the medium comprising (g/L): tryptone 10, yeast extract 5, glucose 10, K_2_HPO_4_ 5, MgSO_4_ 1, trisodium citrate 0.5 and soybean phospholipids 7.5; pH 7.5 [8]. *Escherichia coli* strains JM109 and DH5α were used as hosts for recombinant vectors and cultured in a LB medium [9]. The vectors pUCm-T (Sangon, Shanghai, China) and pPIC9K (Invitrogen, San Diego, CA, USA) were used for gene cloning and expression, respectively. *P. pastoris* GS115 was used as host for expression of *Pcmdl*.

### Cloning of the cDNA Gene Encoding PcMdl

The total RNA was extracted from *P. cyclopium* mycelia using a RNA Extraction Kit (Sangon, China) according to the method as described previously [10]. Based on the information on the genomic DNA sequence of a mono- and diacylglycerol lipase gene (*mdlC*) from *P. cyclopium* PG37 (GenBank: AF288219) and the bioinformatics analysis of signal peptide, a pair of specific primers were designed to amplify the cDNA gene, *Pcmdl*, encoding a mature peptide (that is, PcMdl). The forward and reverse primers, synthesized by Sangon (China), were MDL-F: 5′-GAATTCGATGTTTCGACCAGCGAAC-3′ with an *Eco*R I site (underlined) and MDL-R: 5′-GCGGCCGCTTAAACCCTCTTGAATGGCAG-3′ with a *Not* I site (underlined), respectively.

PcMdl-encoding cDNA gene, *Pcmdl*, was obtained using both the reverse transcription-PCR and nested PCR techniques. An Oligo dT-M13 Primer M4, 5′-GTTTTCCCAGTCACGAC(dT_18_)-3′ provided by RNA PCR Kit (TaKaRa, Dalian, China), was employed for reverse transcription of the first-strand cDNA from *P. cyclopium* total RNA. Using the resulting first-strand cDNA as the template, the first-round amplification of nested PCR was carried out with the primers MDL-F and M13 Primer M4 (identical to Oligo dT-M13 Primer M4 except Oligo dT_18_). Conditions for the first-round PCR were as follows: a starting denaturation for 3 min at 94°C; 30 cycles of for 30 s at 94°C, 30 s at 52°C, 60 s at 72°C; and a final elongation for 10 min at 72°C. Then, *Pcmdl* was amplified from the first-round PCR product by the second-round amplification of nested PCR with the primers MDL-F and MDL-R under the same conditions as stated above, except 55°C instead of 52°C in 30 cycles. The amplified target sequence was inserted into pUCm-T vector, and transformed into *E. coli* JM109. The proper recombinant T-vector, designated as pUCm-T-*Pcmdl*, was confirmed by DNA sequencing.

### Analysis of the Primary and 3-D Structures of PcMdl

The bioinformatics analysis of the signal peptide of PcMdl was carried out using the SignalP 3.0 program (http://www.cbs.dtu.dk/services/SignalP/). The physicochemical properties of PcMdl were identified using the Protparam program (http://au.expasy.org/tools/protparam.html). The homology search for lipase primary structures was performed using the BLAST server at NCBI website (http://www.ncbi.nlm.nih.gov/). The multiple homology alignment of the amino acid sequences among lipases was accomplished using the ClustalW2 program (http://www.ebi.ac.uk/Tools/msa/ClustalW2) and DNAMAN 6.0 software (http://dnaman.software.informer.com). The putative N-linked glycosylation site of PcMdl was located using the NetNGlyc program 1.0 (http://www.cbs.dtu.dk/services/NetNGlyc/). The 3-D structure of PcMdl was homologically modeled using the MODELLER 9.9 program (http://salilab.org/modeller/) based on the known crystal structure of a lipase from *P. camembertii* (PDB: 1TIA). The modeled 3-D structure of PcMdl was visualized using the PyMOL software (http://pymol.org).

### Expression of *Pcmdl* in *P. pastoris*

The gene *Pcmdl* was excised from pUCm-T-*Pcmdl* and inserted into the pPIC9K vector at the *Eco*R I and *Not* I sites. The resulting pPIC9K-*Pcmdl* was linearized with *Sal* I, and transformed into *P. pastoris* GS115 by electroporation using a Gene Pulser apparatus (Bio-Rad, Hercules, CA, USA) according to the manufacturer's instruction. The *P. pastoris* transformants were screened by an MD plate and then a set of increasing concentrations of G418-containing (up to 4.0 mg/mL) YPD plates in turn. A negative control strain (*P. pastoris* GSC) was constructed by introducing only the vector pPIC9K by electroporation into *P. pastoris* GS115. The heterologous expression of *Pcmdl* in *P. pastoris* GS115 was performed followed the instruction of Multi-Copy Pichia Expression Kit (Invitrogen, USA) with slight modifications. Each single colony of the *P. pastoris* transformants was inoculated into a 30 mL of BMGY medium in a 250 mL flask, and cultured at 30°C on a rotary incubator (220 rpm) until the OD_600_ reached 2–4. Then, the yeast cells were harvested by centrifugation, resuspended in a 30 mL of BMMY medium, and induced for rePcMdl expression by adding methanol to a final concentration of 1.0% (v/v) at 24 h intervals at 30°C for 72 h.

### Enzyme Activity and Protein Assays

A quantitative assay for rePcMdl activity was performed according to the method described previously with modifications [8]. In brief, 1 vol of 1,2-dibutyrin (Sangon, China) and 7 vol of 1.0% (w/v) polyvinyl alcohol in 50 mM Na_2_HPO_4_–NaH_2_PO_4_ buffer (pH 7.5) were mixed with a blender (10,000 rpm) till homogeneity was achieved. Then, 9 mL of this emulsified 1,2-dibutyrin was incubated with 1 mL of suitably diluted enzyme at 30°C for 10 min, and terminated by the addition of 15 mL ethanol. The amount of released free butyric acid was measured by titration using 50 mM NaOH. One unit (U) of lipase activity was defined as the amount of enzyme liberating 1 µmol of fatty acid per minute under the standard assay conditions (at pH 7.5 and 30°C for 10 min). Corresponding to U/mg, it was defined as the active amount (U) per milligram of purified lipase protein. To confirm the substrate selectivity of PcMdl, 1-monobutyrin (Sigma, St. Louis, MO, USA) and tributyrin (Sangon, China) were pretreated and used as substrate like 1,2-dibutyrin in quantitative assay for rePcMdl activity. All data of enzyme activities or relative ones were expressed as the mean ± standard deviation (SD) from three independent experiments or parallel measurements.

Sodium dodecyl sulfate polyacrylamide gel electrophoresis (SDS-PAGE) was carried out as reported previously on a 12.5% gel [11]. Coomassie Brilliant Blue R-250 (Sigma, USA) and Quantity One software were used for visualizing and estimating the apparent molecular weights of the separated proteins, respectively. The BCA-200 Protein Assay Kit (Pierce, Rockford, IL, USA) was applied for protein concentration measuring. The expressed rePcMdl was purified by a simple combination of ammonium sulfate precipitation, ultrafiltration and Sephadex G-75 gel filtration [12].

### Determination of Temperature Optimum and Stability of rePcMdl

The temperature optimum of rePcMdl was measured under the standard lipase activity assay conditions, except reaction temperatures of 20–50°C. To evaluate its temperature stability, aliquots of rePcMdl were incubated in the absence of substrate at different temperatures (20–45°C), respectively, for 1.0 h. To avoid effect of temperature on the pH value, the 50 mM Na_2_HPO_4_–NaH_2_PO_4_ buffer had been accurately adjusted to pH 7.50 at different temperatures (20–50°C). The residual activity of rePcMdl was measured under the standard assay conditions. In this work, the thermostability was defined as the temperature, at or below which the residual activity of rePcMdl was over 85% of its initial activity.

### Determination of pH Optimum and Stability of rePcMdl

The pH optimum of rePcMdl was determined using the emulsified 1,2-dibutyrin prepared with 50 mM Na_2_HPO_4_–NaH_2_PO_4_ buffer (pH 6.5–8.0) and glycine–NaOH buffer (pH 8.5–9.0), at the optimum temperature for 10 min. To estimate its pH stability, aliquots of rePcMdl were incubated at 30°C and at different pH values (50 mM Na_2_HPO_4_–NaH_2_PO_4_ buffer, pH 6.0–8.0; 50 mM glycine–NaOH buffer, pH 8.5–10.0) for 1.0 h. The pH range over which the retained activity of rePcMdl was more than 85% of its initial activity was defined as the range of its pH stability.

### Effects of Metal Ions and EDTA on rePcMdl Activity

To estimte its tolerance to metal ions (Li^+^, Na^+^, K^+^, Mg^2+^, Zn^2+^, Ca^2+^, Mn^2+^, Hg^2+^, Cu^2+^, Fe^2+^, and Fe^3+^) and EDTA, aliquots of rePcMdl were incubated with an array of metal ions and EDTA, respectively, at a final concentration of 5.0 mM in 20 mM 4-(2-hydroxyethyl)-1-piperazineethanesulfonic acid (HEPES) buffer (pH 7.5) at 30°C for 1.0 h. The residual activity of rePcMdl was determined under the standard assay conditions except in 20 mM HEPES buffer (pH 7.5). The enzyme solution without adding any additive was used as the control.

### Lipid Hydrolysis by rePcMdl and Triacylglycerol Lipase

Two triacylglycerol lipases, a neutral *Candida antarctica* lipase B (CALB) and an alkaline *P. cyclopium* lipase (Lip I ) which were purchased from Sigma (USA) and produced by the method reported previously [7] in turn, were used for tests of olive oil hydrolysis with rePcMdl. The hydrolysis by CALB and rePcMdl was carried out at pH 7.5 and 30°C, while by Lip I and rePcMdl was at pH 8.5 and 25°C. An emulsified olive oil was used as the substrate in the hydrolysis experiments. Briefly, 1 vol of olive oil and 3 vol of 3% (w/v) polyvinyl alcohol were mixed well with a blender (10,000 rpm) to form the emulsified substrate. Aliquots of 20 mL 50 mM Na_2_HPO_4_–NaH_2_PO_4_ (pH 7.5) or glycine–NaOH (pH 8.5) buffer were mixed well with 8 mL emulsified substrate and 1 mL diluted rePcMdl, triacylglycerol lipase or two lipases mixture with appropriate activity units, respectively. The mixture of substrate and lipase(s) was incubated at 25 or 30°C for 2 h on a magnetism mixer with 200 rpm agitation, meanwhile titration was performed with 50 mM NaOH in a pH-stat to keep pH 7.5 or 8.5. The heat inactivated lipase instead of the active one was used as the control. The total volume of the used NaOH measured at 5 min intervals for first hour and at 10 min intervals for the next hour had a positive correlation with the amount of free fatty acids released from olive oil.

### 3-D Structure of PcMdl in an Open Conformation

The primary sequence of PcMdl shares a 98.6% identity with the MdlA from *P. camembertii* (PDB: 1TIA), which is in a closed conformation. In order to provide insight into the responsibility for substrate selectivity of PcMdl, its open conformation was homologically modeled using the crystal structure of *T. lanuginosa* lipase (PDB: 1GT6) as the template on the MODELLER 9.9 program. The modeled 3-D structure of PcMdl was visualized using the PyMOL software.

### Molecular Docking Simulation

A triacylglycerol analogue, Rc-(Rp, Sp)-1,2-dioctylcarbamoyl-glycero-3-o-octylphosphonate, was extracted from the crystal structure of *Pseudomonas aeruginosa* lipase (PDB: 1EX9). A diacylglycerol analogue, Rc-(Rp, Sp)-2-monooctylcarbamoyl-glycero-3-o-octylphosphonate, was derived from the triacylglycerol analogue mentioned above by removing the *sn*-1 moiety. The interaction of PcMdl with a tri- or diacylglycerol analogue was predicted by molecular docking simulation using the AutoDock 4.2 program (http://autodock.scripps.edu) to locate the most suitable binding position and orientation.

### Molecular Dynamics Simulations

In order to fully relax the steric clashes in PcMdl-analogue docked complexes, molecular dynamics (MD) simulations were carried out by the Discovery Studio ver. 3.5 (http://accelrys.com/). The CHARMm force field was used for MD simulations. All structures were inserted into an octahedral box with explicit solvent and simulated with periodic boundary conditions. Then the systems were neutralized by insertion of counter ions. First energy minimization was performed by 2,000 steps of steepest descent algorithm with a RMS gradient of 0.1 kcal/mol. Second energy minimization was carried out by 20,000 steps of conjugate gradient algorithm with a RMS gradient of 0.0001 kcal/mol. The following simulations were overall conducted using Particle Mesh Ewald electrostatics. Heating and cooling simulations were run at an initial and a target temperature, 50 and 300 K, respectively, for 4,000 steps (0.001 ps per step). Equilibration simulations were undergone at a target temperature 300 K for 4,000 steps (0.001 ps per step). Production simulations were worked at a target temperature 300 K for 30,000 steps (0.001 ps per step). The optimized 3-D structure of PcMdl-substrate analogue was visualized using the PyMOL software.

## Results and Discussion

### Cloning of the cDNA Gene *Pcmdl*

Using the first-strand cDNA transcribed reversely from the *P. cyclopium* PG37 total RNA as the template, an about 1.1-kb clear band and several faint bands were amplified by the first-round PCR. On the basis of the principle of nested PCR technique, each band was gel-purified, and subjected to the second-round PCR. As a result, an about 850-bp specific band was amplified only using a 1.1-kb band as the template. The nucleotide sequence of the cDNA gene *Pcmdl* encoding PcMdl has been deposited in the GenBank database under accession no. HM135194.

### Analysis of the Primary and 3-D Structures of PcMdl

**Figure 1:**
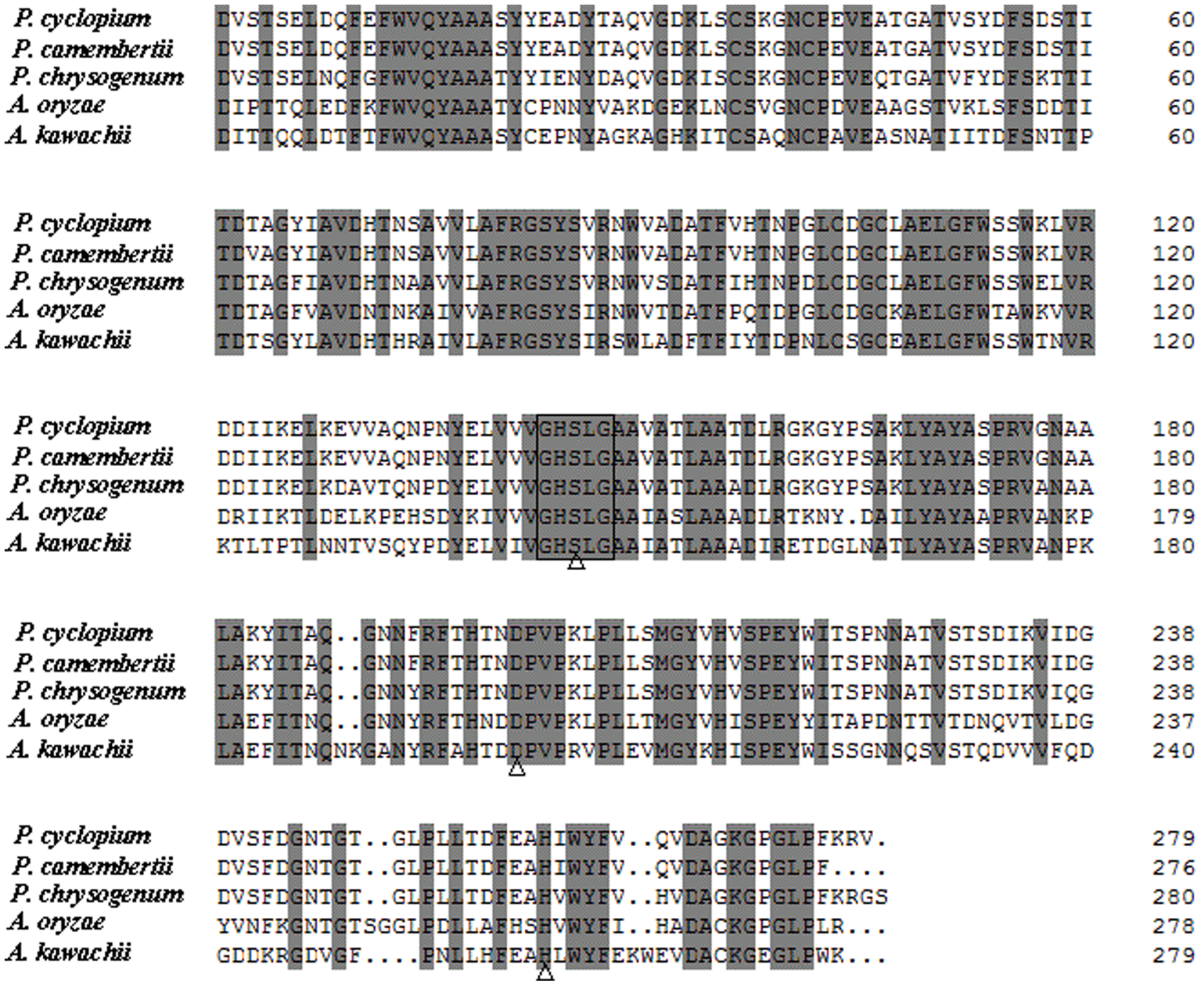
Multiple alignment of primary structures between PcMdl and four homologous mono- and diacylglycerol lipases. The homology identities of PcMdl from *Penicillium cyclopium* (in this work) with other four mono- and diacylglycerol lipases from *Penicillium camembertii* (GenBank: AAB26004), *Penicillium chrysogenum* (GenBank: XP_002567712), *Aspergillus oryzae* (GenBank: XP_001823459), and *Aspergillus kawachii* (GenBank: GAA92588) are 98.6%, 89.0%, 63.7%, and 58.3%, respectively. The identical amino acid residues in five lipases are marked in grey background. A pentapeptide G-H-S-L-G is strictly conserved (boxed), corresponding with the classical motif G-X-S-X-G (X: arbitrary amino acid residue). The triangles below three letters indicate the conserved catalytic triad (Ser, Asp, His). The cDNA sequence encoding PcMdl has been deposited to GenBank under accession no. HM135194.

The cloned cDNA gene *Pcmdl* is exactly 840 bp in length, encoding a mature peptide (PcMdl) of 279 amino acids (Fig. 1, line 1). The theoretical molecular weight and isoelectric point (pI) of PcMdl were calculated to be 30,181 Da and 4.81, respectively. The homology alignment between two amino acid sequences using the DNAMAN 6.0 software displayed that the identities of PcMdl from *P. cyclopium* (in this work) with other four mono- and diacylglycerol lipases from *P. camembertii* (GenBank: AAB26004), *Penicillium chrysogenum* (GenBank: XP_002567712), *A. oryzae* (GenBank: XP_001823459), and *Aspergillus kawachii* (GenBank: GAA92588), were 98.6%, 89.0%, 63.7%, and 58.3%, respectively. Moreover, the multiple homology alignment among the five lipase sequences showed that a pentapeptide G-H-S-L-G (located at a site from Gly^143^ to Gly^147^) was strictly conserved (Fig. 1), corresponding with the classical motif G-X-S-X-G (X: arbitrary amino acid residue) of almost all lipases or esterases reported thus far [13], [14]. While, the most conserved three amino acid residues, Ser^145^, Asp^199^, and His^259^ (numbered by PcMdl), constitute a typical catalytic triad of lipases. All these primary structure features implied that PcMdl is a member of the lipase family.

**Figure 2:**
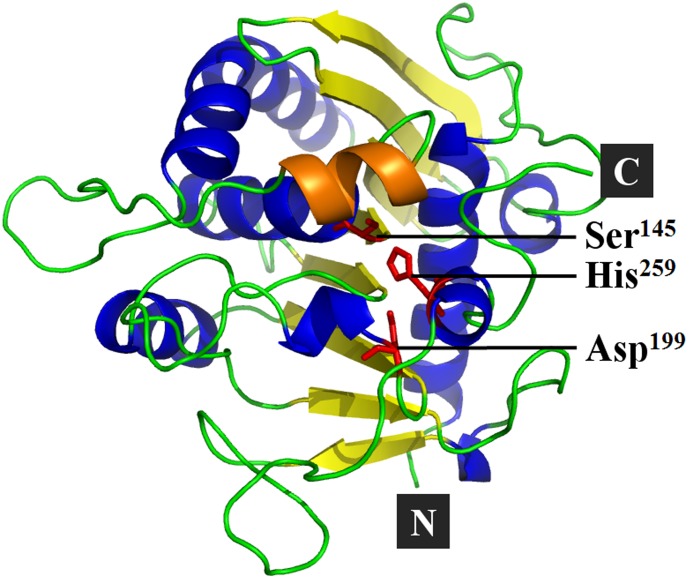
The 3-D structure model of PcMdl. The 3-D structure of PcMdl was predicted by homology modeling based on the known crystal structure of the *Penicillium camembertii* MdlA (PDB code: 1TIA), which shares a 98.6% identity with PcMdl in the primary structure. The modeled 3-D structure displays a globular shape, which is composed of one eight-stranded β-sheet, three long and several short α-helices, and a catalytic triad Ser^145^-Asp^199^-His^259^.

Based on the known crystal structure of the *P. camembertii* (PDB: 1TIA), which shares a 98.6% identity with PcMdl in the primary structure, the 3-D structure of PcMdl was predicted by homology modeling in the closed conformation as shown in Fig. 2. The modeled 3-D structure displays a globular shape, which is composed of one eight-stranded β-sheet, three long and several short α-helices, and the catalytic triad Ser^145^-Asp^199^-His^259^. Although there are wide differences in their primary structures and biochemical properties, PcMdl and the majority of lipases share a similar core topology of the 3-D structure, well known as the α/β hydrolase fold [15].

### Expression of *Pcmdl* in *P. pastoris*

The protein expression level was not directly proportional to the concentration of geneticin G418 [11]. For this reason, 45 transformants separately resistant to 1.0, 2.0, and 4.0 mg/mL of G418, numbered as *P. pastoris* GSM1-1 to GSM1-15, GSM2-1 to GSM2-15, and GSM4-1 to GSM4-15, were picked out for flask expression tests. *P. pastoris* GS115 transformed with pPIC9K vector instead of pPIC9K-*Pcmdl*, numbered as *P. pastoris* GSC, was used as the negative control. After *P. pastoris* transformants were induced by 1.0% methanol for 72 h, activities and proteins of the expressed rePcMdl in the cultured supernatants were assayed. Among all transformants tested, one transformant expressing the highest rePcMdl activity of 40.5 U/mL, *P. pastoris* GSM4-2, was selected and used for subsequent studies. As a negative control strain, *P. pastoris* GSC expressed no lipase activity in the cultured supernatant.

### Protein Assays of the Expressed rePcMdl

**Figure 3:**
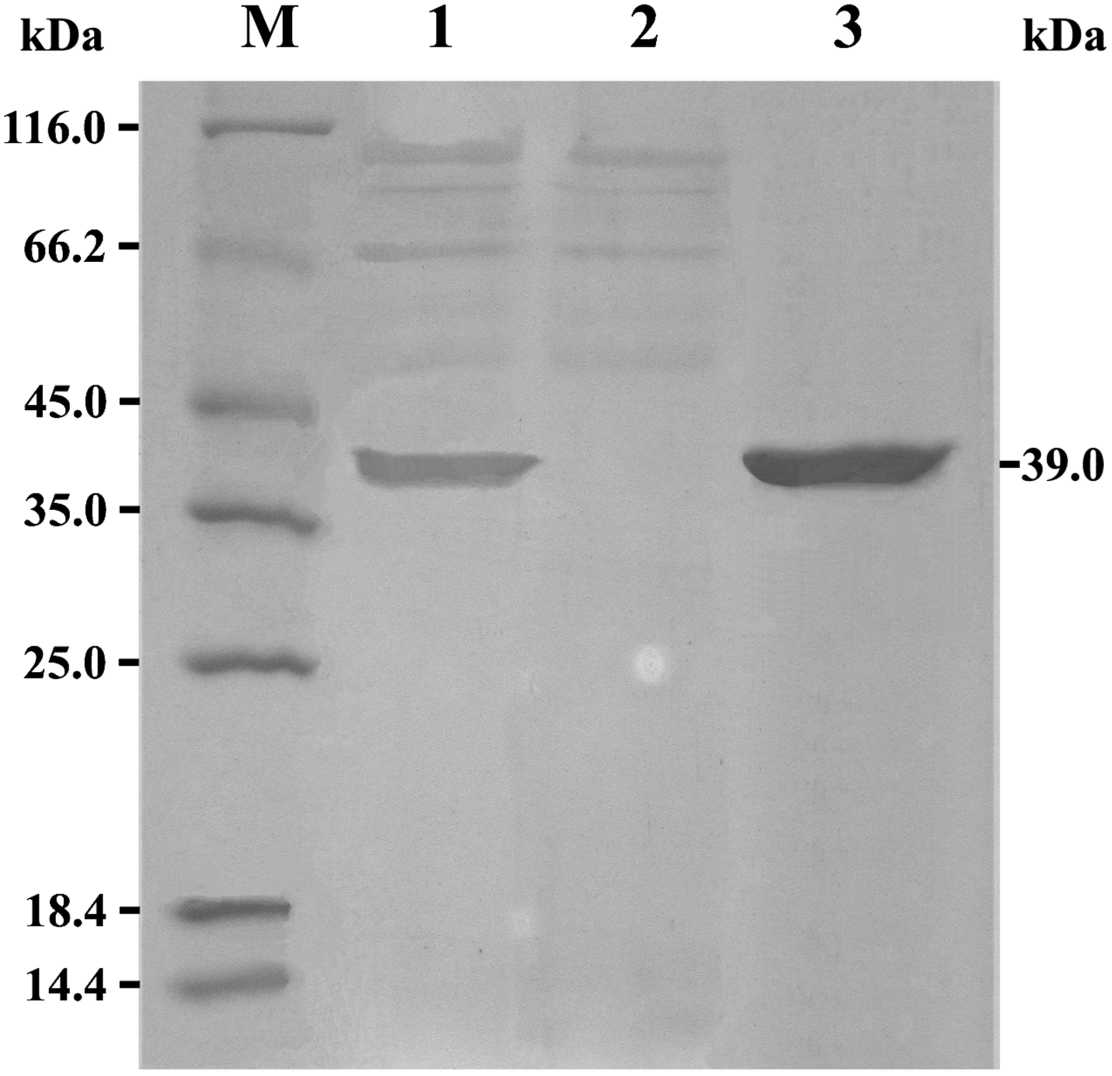
SDS-PAGE analysis of rePcMdl secreted by *Pichia pastoris* GSM4-2. Lane M, protein marker; lane 1, the culture supernatant of the positive transformant (GSM4-2); lane 2, the culture supernatant of the negative control (GSC); and lane 3, the purified rePcMdl.

It was reported that the purities of the recombinant *Aspergillus sulphureus* β-mannanase and *Aspergillus usamii* xylanase AuXyn11D expressed in *P. pastoris* X-33 and GS115 were 97% and 90%, respectively [16], [17]. In this work, by protein band-scanning, more than 86% of the total supernatant protein was the secreted rePcMdl from *P. pastoris* GSM4-2 (Fig. 3, lane 1). Therefore, the expressed rePcMdl was purified to homogeneity by a simple combination of ammonium sulfate precipitation, ultrafiltration and Sephadex G-75 gel filtration (Fig. 3, lane 3).

SDS-PAGE analysis of the purified rePcMdl displayed a distinctive protein band (Fig. 3, lane 3). The apparent molecular weight (approximate 39 kDa) of rePcMdl was similar to that of the mono- and diacylglycerol lipase purified from *P. camembertii* [2], but much larger than its theoretical weight (30,181 Da). *P. pastoris* enables expressed proteins to undergo some post-translational modifications, such as the exclusion of signal peptide [18], assembly of disulfide bridges and glycosylation of the mature peptide. The bioinformatics analysis of PcMdl amino acid sequence using the NetNGlyc program displayed that there is one putative N-linked glycosylation site (N^225^-A^226^-T^227^). Simultaneously, the carbohydrate content of the purified rePcMdl was assayed to be 12.3% using the phenol-sulfuric acid method [19]. These analytical results implied that the increased molecular weight of rePcMdl was caused by N-glycosylation [12].

### Partial Enzymatic Properties of rePcMdl

**Figure 4:**
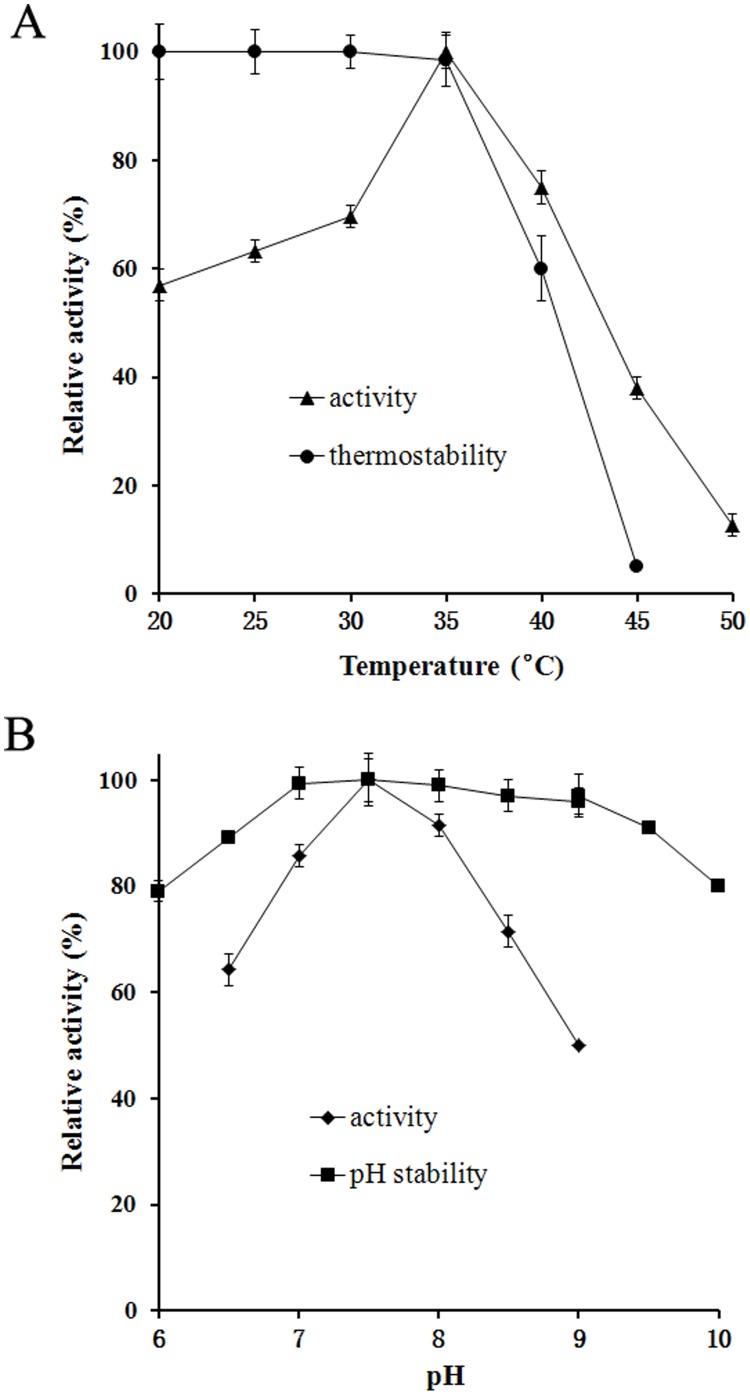
Effects of temperature and pH on rePcMdl. (A) The temperature optima and thermostability of rePcMdl. The temperature optimum (100% relative activity) of rePcMdl, measured at pH 7.5, was 35°C. Incubated at different temperatures for 1.0 h, the residual activity of rePcMdl retained over 85% of its original activity at 35°C or below, but declined rapidly over 35°C. (B) The optimal pH and pH stability of rePcMdl. The purified rePcMdl exhibited higher relative activities over a pH range of 7.0–8.0 (measured at 35°C). Its pH optimum (100% relative activity) was at pH 7.5. Incubated at different pH values (6.0–10.0) for 1.0 h, rePcMdl was highly stable (more than 85%) over a broad pH range of 6.5–9.5.

**Figure 5:**
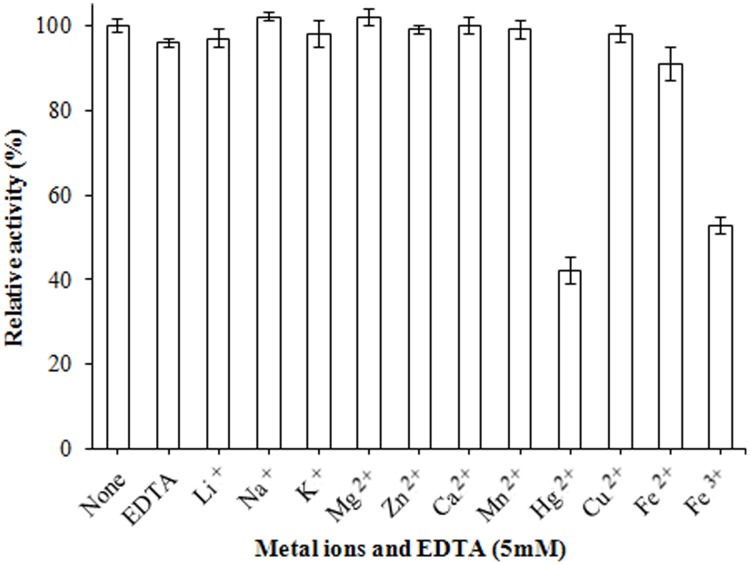
Effects of various metal ions and EDTA on rePcMdl activity. The rePcMdl activity was not significantly affected by metal ions that were tested or EDTA, but was inhibited by Hg^2+^ and Fe^3+^.

The specific activity of the purified rePcMdl, towards 1,2-dibutyrin under the standard assay conditions, was 344.9 U/mg. The temperature optimum (100% relative activity) of rePcMdl at pH 7.5 was 35°C (Fig. 4A). It was similar to that of the mono- and diacylglycerol lipase (MDL) of *P. camembertii* [6], but 10°C higher than that of the triacylglycerol lipase (Lip I ) from *P. cyclopium* PG37 [7]. Incubated at different temperatures for 1.0 h, the residual activity of rePcMdl retained over 85% of its original activity at 35°C or below, but declined rapidly over 35°C (Fig. 4A). The purified rePcMdl exhibited higher relative activities over a pH range of 7.0–8.0 (measured at 35°C). Its pH optimum (100% relative activity) was at pH 7.5 (Fig. 4B), which was similar to that of the MDL of *P. camembertii* and much lower than that (pH 10.5) of Lip I. Incubated at different pH values (6.0–10.0) for 1.0 h, rePcMdl was highly stable (more than 85%) over a broad pH range of 6.5–9.5 (Fig. 4B). Incubated with an array of metal ions and EDTA, respectively, the activity of rePcMdl was not significantly affected by most metal ions tested and EDTA, but inhibited by Hg^2+^ and Fe^3+^ (Fig. 5). The rePcMdl was confirmed to be strictly specific to 1-monobutyrin (189.3 U/mg) and 1,2-dibutyrin (344.9 U/mg), but not tributyrin (0 U/mg).

### Lipid Hydrolysis by rePcMdl and Triacylglycerol Lipase

**Figure 6:**
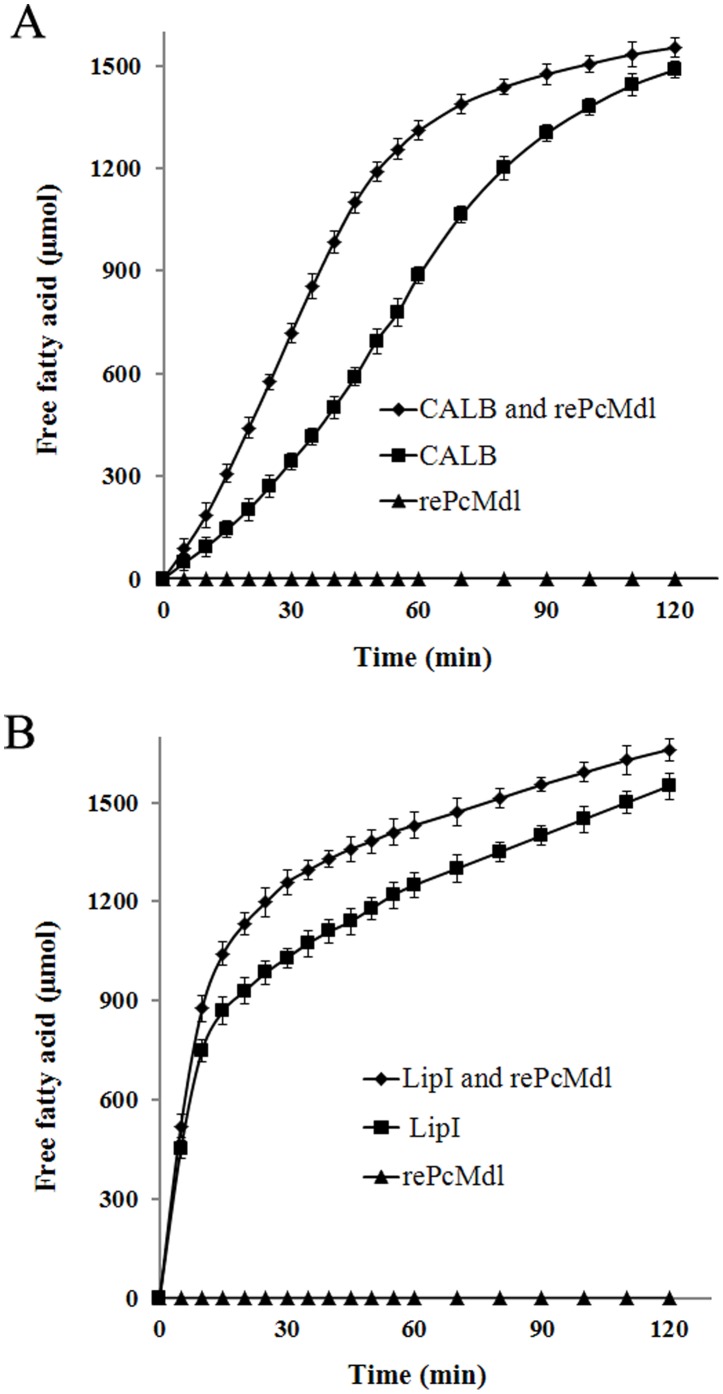
The lipid hydrolysis by rePcMdl and triacylglycerol lipase. (A) The olive oil hydrolysis carried out at pH 7.5 and 30°C by *C. antarctica* lipase B (CALB) and rePcMdl, (B) while at pH 8.5 and 25°C by *P. cyclopium* PG37 lipase I (Lip I) and rePcMdl. The rePcMdl could not hydrolyze olive oil without help from CALB or Lip I. CALB or Lip I together with rePcMdl had bigger effect on olive oil hydrolysis than CALB or Lip I alone.

The rePcMdl could not hydrolyze olive oil without help from CALB or Lip I (Fig. 6). CALB or Lip I together with rePcMdl had bigger effect on olive oil hydrolysis than CALB or Lip I alone. By lipase catalyzing, olive oil was hydrolyzed and mainly releasing oleic acids [20]. The shape of the hydrolysis curves mainly depended on the characteristic of the triacylglycerol lipase, CALB or Lip I .

### Molecular Basis for Substrate Selectivity of PcMdl

**Figure 7:**
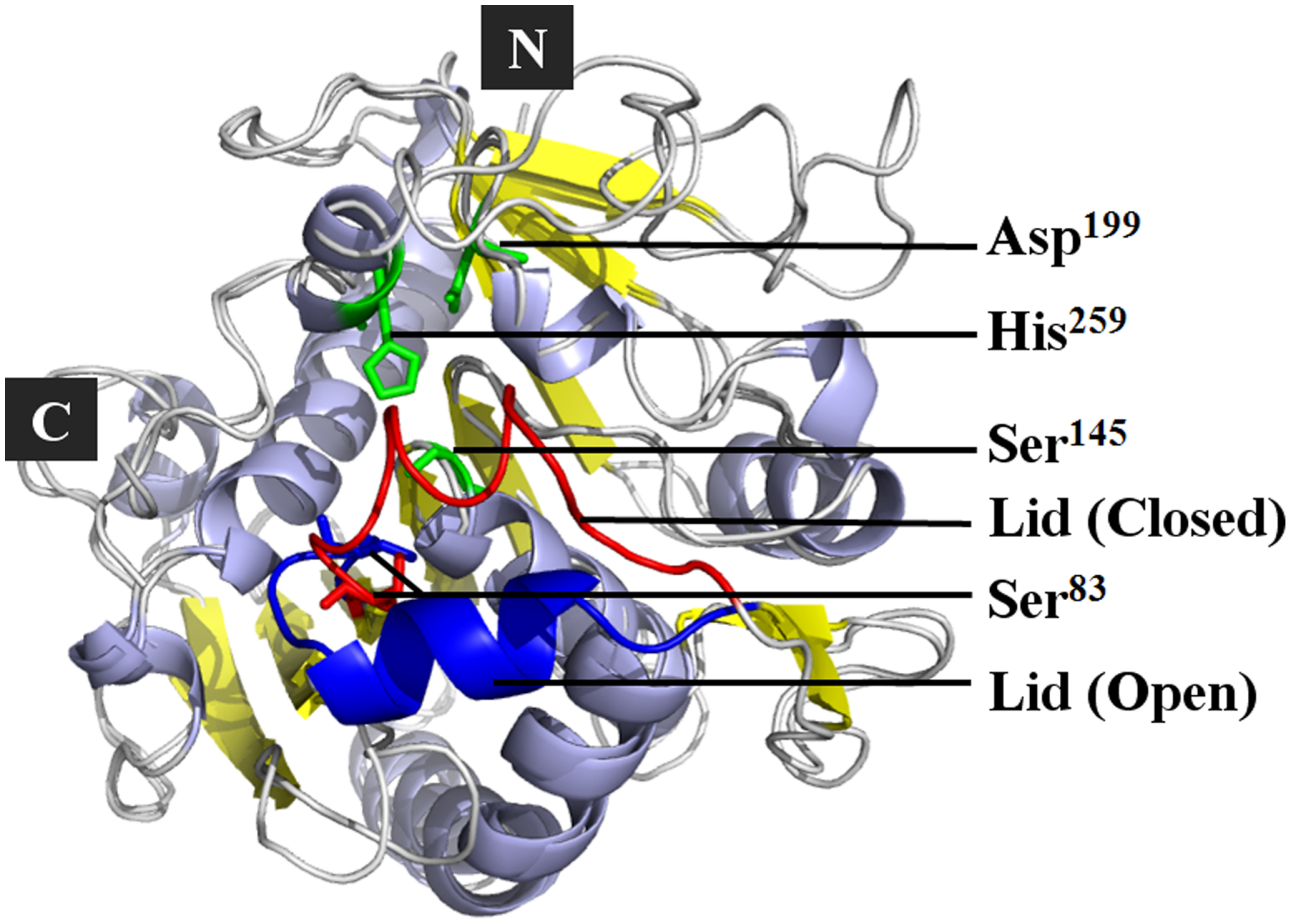
The 3-D structure superimposition of PcMdls in a closed and an open conformations. The difference between the open and closed conformations of PcMdls mainly exists in lid orient (highlighted in blue and red for open and closed, respectively). The α-helix form lid in the open conformation uncovers the catalytic center and leads to the catalytic triad Ser^145^-Asp^199^-His^259^ exposing to the solvent. The residue Ser^83^ together with the Ser^145^ constitutes the oxyanion hole which stabilizes the tetrahedral intermediates.

**Figure 8:**
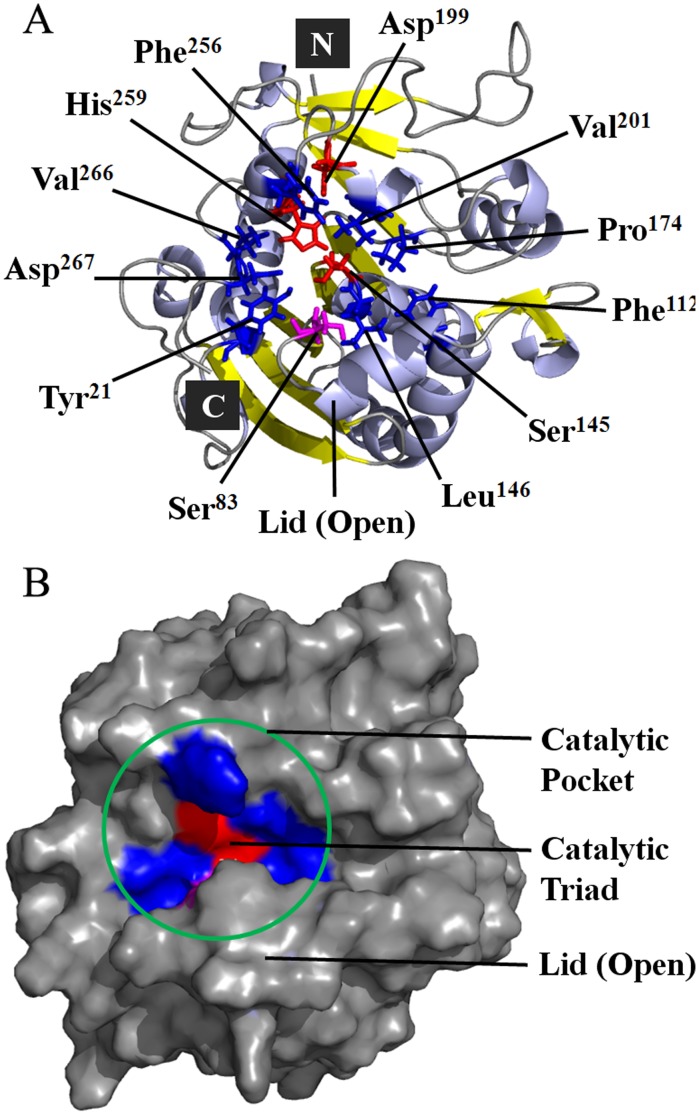
The catalytic pocket of PcMdl in an open conformation. (A) Cartoon view of the modeled PcMdl. The catalytic pocket, organized by residues Tyr^21^, Phe^112^, Leu^146^, Pro^174^, Val^201^, Phe^256^, Val^266^, and Asp^267^, exposes to the solvent with the lid opening. (B) Surface view of the modeled PcMdl. The catalytic pocket and catalytic triad, highlighted in blue and red, respectively, exposes to the solvent.

The active PcMdl in the open conformation was modeled with the lid in α-helix form, uncovered the catalytic center and led to the catalytic triad Ser^145^-Asp^199^-His^259^ exposing to the solvent (Fig. 7). The difference between the open (modeled structure) and closed (crystal structure) conformations of PcMdls mainly existed in lid orient. In the open conformation, the residue Ser^83^ together with the Ser^145^ (Fig. 7), constituted the oxyanion hole which stabilizes the tetrahedral intermediates. The catalytic pocket, organized by residues Tyr^21^, Phe^112^, Leu^146^, Pro^174^, Val^201^, Phe^256^, Val^266^, and Asp^267^, exposed to the solvent with the lid opening (Fig. 8). All of the above made the modeled open conformation be reliable [4], .

**Figure 9:**
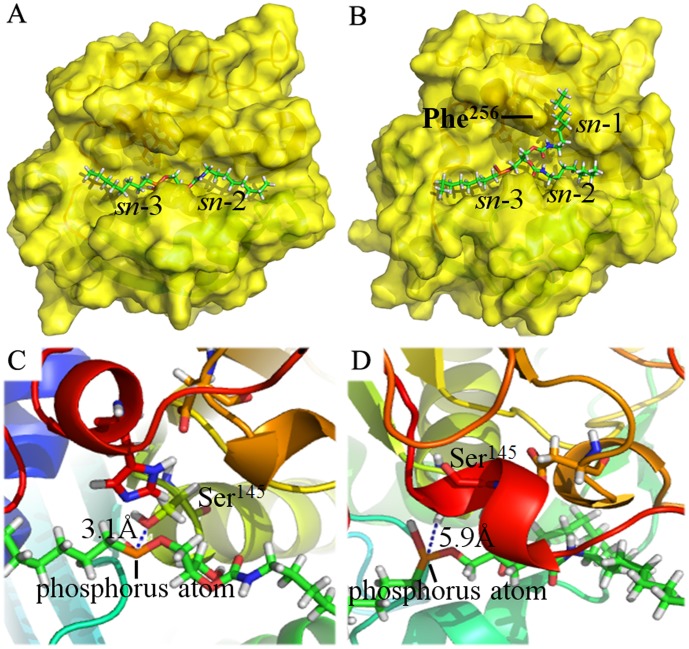
The 3-D structures of PcMdl-analogue complexes. (A) Surface view of the optimized PcMdl-diacylglycerol analogue. (B) Surface view of the optimized PcMdl-triacylglycerol analogue. (C) A close-up view showing the distance from diacylglycerol analogue phosphorus atom (substitution of carboxyl carbon) to O_γ_ of Ser^145^ in PcMdl. (D) A close-up view showing the distance from triacylglycerol analogue phosphorus atom (substitution of carboxyl carbon) to O_γ_ of Ser^145^ in PcMdl.

After MD simulations, PcMdl-analogue docked complexes fully relaxed the steric clashes. In the optimized PcMdl-diacylglycerol analogue complex (Fig. 9A and C), the distance between phosphorus atom (substitution of carboxyl carbon) and O_γ_ of Ser^145^ is 3.1 Å, while in the optimized PcMdl-triacylglycerol analogue complex (Fig. 9B and D) it is 5.9 Å. A smooth reaction of lipid hydrolysis requires that a distance between O_γ_ of serine in catalytic triad and carboxyl carbon of substrate is no more than 4.0 Å [24], [25]. The Phe^256^ projects its side chain towards the substrate binding groove and makes the *sn*-1 moiety difficult to insert in. Furthermore, *sn*-1 moiety prevents the phosphorus atom from getting to the O_γ_ of Ser^145^. The substrate selectivity mechanism of PcMdl differs from this of Mdl from *M. globosa* despite the steric hindrance effect [4]. In the *M. globosa* Mdl, the Phe^278^ and residue at the opposite side, Leu^103^, forms a bridge-like obstacle and restricts two fatty acid chains fitted in tunnel or groove. In PcMdl, only one residue, Phe^256^, plays an important role in substrate selectivity.

### Conclusions

In this work, *Pcmdl* which encodes the mono- and diacylglycerol lipase from *P. cyclopium* PG37 was cloned and expressed in *P. pastoris* GS115. The recombinant PcMdl was characterized and confirmed to be strictly specific to mono- and diacylglycerol, but not triacylglycerol. Stereographic view of PcMdl docked with substrate analogue indicated that the residue Phe^256^ plays an important role in conferring the substrate selectivity. These results should provide a basis for molecular engineering of PcMdl and expand its applications in industries.
