# Correction: A Unique Mono- and Diacylglycerol Lipase from *Penicillium cyclopium*: Heterologous Expression, Biochemical Characterization and Molecular Basis for Its Substrate Selectivity

**DOI:** 10.1371/journal.pone.0109496

**Published:** 2014-09-26

**Authors:** The PLOS ONE Staff

There is an error in the affiliation for the first author. Zhong-Biao Tan is also affiliated with: Jiangsu Provincial Engineering Laboratory for Biomass Conversion and Process Integration, School of Life Science and Chemical Engineering, Huaiyin Institute of Technology, Huai'an, China
